# The Late Dr. Reginald Farrar

**Published:** 1922-02

**Authors:** 


### THE LATE DR. REGINALD FARRAR.

WE regret to hear of the death of Dr. Reginald Farrar, M.A., M.D.(Oxon.), on December 29. His fatal illness (believed to be typhus) was contracted at Moscow, where he was assisting Dr. Nansen in organising arrangements for famine relief in Russia under the auspices of the League of Nations and the League of Red Cross Societies.

Son of Dean Farrar, Dr. Reginald Farrar was born in 1861, and educated at Marlborough and Oxford. He studied medicine at St. Bartholomew's Hospital, where he served as House Surgeon to Mr. (afterwards Sir) William Savory. After a few years in general practice at Stamford in Lincolnshire he turned his attention to public health medicine. He served in 1899—1900 under the Government of India in plague and famine work, and in 1903 was appointed a Medical Inspector of the Local Government Board. His reports on the Lodging and Accommodation of Navvies, Hop-pickers, Pea-pickers and Fruit-pickers (1906-1908) illustrate the intimate knowledge he possessed of these classes of workers. In 1911 he acted as British representative on the International Plague Commission in Manchuria, and instituted inquiries for the Local Government Board in China and Siberia respecting pork and bacon exported to England. In 1914 he was engaged in the examination of refugees in Belgium, receiving the Order of Chevalier de la Couronne from the King of the Belgians in recognition of his services. As a temporary Major R.A.M.C. he served as Specialist Sanitary Officer at Malta (1915-16), and later was transferred to the Colonial Office for special work, undertaking the duties of Medical Officer of Health at Freetown, Sierra Leone (1918-19). In 1920 he visited Austria and Hungary in connection with the reception into England of children from the famine area of these countries.

